# An Immune-Related Gene Pairs Signature Predicts Prognosis and Immune Heterogeneity in Glioblastoma

**DOI:** 10.3389/fonc.2021.592211

**Published:** 2021-04-13

**Authors:** Nijia Zhang, Ming Ge, Tao Jiang, Xiaoxia Peng, Hailang Sun, Xiang Qi, Zhewei Zou, Dapeng Li

**Affiliations:** ^1^Department of Pediatric Neurosurgery, Beijing Children’s Hospital, Capital Medical University, National Center for Children’s Health, Beijing, China; ^2^Department of Neurosurgery, Beijing Tiantan Hospital, Capital Medical University, Beijing, China; ^3^Department of Neurosurgery, Beijing Neurosurgical Institute, Capital Medical University, Beijing, China; ^4^Clinical Epidemiology and Evidence-based Medicine Center, Beijing Children’s Hospital, Capital Medical University, National Center for Children’s Health, Beijing, China

**Keywords:** prognosis, immune heterogeneity, immune-related gene pairs signature, glioblastoma, immunotherapy

## Abstract

**Purpose:**

Glioblastoma is one of the most aggressive nervous system neoplasms. Immunotherapy represents a hot spot and has not been included in standard treatments of glioblastoma. So in this study, we aim to filtrate an immune-related gene pairs (IRGPs) signature for predicting survival and immune heterogeneity.

**Methods:**

We used gene expression profiles and clinical information of glioblastoma patients in the TCGA and CGGA datasets, dividing into discovery and validation cohorts. IRGPs significantly correlative with prognosis were selected to conduct an IRGPs signature. Low and high risk groups were separated by this IRGPs signature. Univariate and multivariate cox analysis were adopted to check whether risk can be a independent prognostic factor. Immune heterogeneity between different risk groups was analyzed *via* immune infiltration and gene set enrichment analysis (GSEA). Some different expressed genes between groups were selected to determine their relationship with immune cells and immune checkpoints.

**Results:**

We found an IRGPs signature consisting of 5 IRGPs. Different risk based on IRGPs signature is a independent prognostic factor both in the discovery and validation cohorts. High risk group has some immune positive cells and more immune repressive cells than low risk group by means of immune infiltration. We discovered some pathways are more active in the high risk group, leading to immune suppression, drug resistance and tumor evasion. In two specific signaling, some genes are over expressed in high risk group and positive related to immune repressive cells and immune checkpoints, which indicate aggression and immunotherapy resistance.

**Conclusion:**

We identified a robust IRGPs signature to predict prognosis and immune heterogeneity in glioblastoma patients. Some potential targets and pathways need to be further researched to make different patients benefit from personalized immunotherapy.

## Introduction

Glioblastoma multiforme (GBM) is the most aggressive and malignant tumor in the central nervous system. The current standard therapy involving tumor resection, radiotherapy and chemotherapy implemented in 2005 and have yet to be modified (1). Despite this conventional treatments, the median survival time for GBM is despondingly 12-18 months (2). The evolvement in genomics and proteomics has made researchers acquire prominent molecular biomarkers, while few lead to a robust and innovative signature on GBM therapy (3). Immune system serves as a defensive mechanism against the formation and progression of tumors. Cancer immunotherapy has attracted attention worldwide owing to remarkable success treating advanced cancers (4). GBM immunotherapy is a research hot spot in recent years. However, majority of patients response to immunotherapy ineffectively (5). So there is an urgent need to develop new biomarkers for guiding individual immunotherapy in the treatment of GBM.

Recently, several studies have developed prognostic signatures to dividing GBM patients into different risk groups according to gene expression profiles (6–8). However, gene expression values measured by different platforms might generate sampling bias (9). Effective analysis of large-scale gene expression inflicts a great computational challenge that requires the use of appropriate methods. In order to eliminate the defects of data normalization and scale in gene expression data processing, some researches have invented a new method, which based on relative sequences of gene expression level, and obtained reliable results (10, 11). To date there are no studies using the new method to differentiate prognosis and immune heterogeneity of GBM patients on account of immune-related genes. So in this study, we aimed to explore prognositic immune-related gene pairs (IRGPs) in GBMs and find potential targets could be used to develop new immunotherapeutic agents.

## Materials and Methods

### Data Resources and Processing

This study was a retrospective study using public data. We obtained gene expression profiles and corresponding intact clinical information on 149 GBM samples in the open-source database named TCGA (https://portal.gdc.cancer.gov/) (12). Via another independent database, the Chinese Glioma Genome Atlas (CGGA) (http://www.cgga.org.cn/), we acquired molecular and clinical information of 374 GBM patients from different platforms (13, 14). For TCGA, the gene expression profile on probe level was transformed into gene symbol level. When multiple same gene symbols exist, the highest expression was selected. All expression data in both datasets were not further standardized during establishment of signature.

### Identification and Verification of Immune-Related Gene Pairs (IRGPs) Signature

In this study, we selected GBM patients in the TCGA dataset as the training group, correspondingly the CGGA cohort as the validation group. Previous articles have involved how to construct IRGPs (11). We obtained 2498 immune-related genes (IRGs) in the ImmPort database (https://immport.niaid.nih.gov) (15). These immune-related genes include 17 immune classifications according to different molecular function, such as antimicrobials, antigen processing and presentation, cytokines, interleukins, natural killer cell cytotoxicity, TNF family receptors. The IRGs owning relatively high variable quantity (measured by median absolute deviation >0.5) were selected across all different platforms (16). Pairwise comparison was undertaken to obtain IRGPs using the gene expression level in one particular sample. In simple terms, a score of IRGP was 1 if IRG 1 was higher than IRG 2; conversely the IRGP score was 0. We abandoned IRGPs with low variations and the rest of IRGPs were optimized to conduct prognostic IRGPs by means of cox regression, log-rank test and multiple lasso regression. Immune-related gene pairs index (IRGPI) was produced in the training cohort by means of lasso penalized cox regression with 10-fold internal cross-validation in the glmnet package (version 3.0-2). A time-dependent receiver operating characteristic (ROC) curve (survivalROC, version 1.0.3) was generated to ensure the optimal cut-off value of IRGPI using overall survival in the TCGA dataset for distinguishing high risk from low risk patients. Between different risk groups, we used log-rank test to evaluate the established model in both the two datasets. Then we assessed whether risk based on this IRGPs signature could be an independent prognostic factor compared with other clinical factors using the univariate and multivariate cox proportional-hazards analysis.

### Estimation and Comparison of the Immune Infiltration Pattern Between Different Risk Groups

We used the RNA-seq data, which include 149 GBM patients from the TCGA database and 139 patients from the same platform in the CGGA cohort, *via* a sample-level enrichment method named single sample gene set enrichment analysis (ssGSEA) in the GSVA package (version 1.34.0) to calculate the relative abundance of 30 immune cells of each patients in two distinct risk groups (17). From previous publications (18, 19), distinct genes which are highly expressed in each cell type were selected to represent immune populations. We used heatmap in the package ComplexHeatmap (20) to present the overall immune infiltration associated with some important mutations and clinical information in two groups. Then we detailedly compared whether average normalized enrichment scores (NES) of immune cells are significantly different between high risk and low risk groups.

### Gene Set Enrichment Analysis (GSEA)

In order to determine the different expression of the same gene between high and low risk groups, we used log2 fold change of average gene expression from two groups. We conducted gene set enrichment analysis with 1000 permutations from clusterProfiler package (version 3.14.3) (21). Hallmark gene sets concerned with this study were downloaded from the Molecular Signature Database (version 7.1) (http://www.broadinstitute.org/gsea/msigdb/collections.jsp) (22). The particular gene sets whose adjusted p-value less than 0.05 were kept as statistically significant pathways.

### Initial Estimate Potential Target Genes

Some genes expressed differently between two groups were extracted in specific pathways which are significant in both cohorts. To determine whether these genes could be potential targets for immunotherapy, we analyzed the relationship between these genes and immune infiltration. Immune checkpoints including PD-L1/CD274 and CTLA4 have been targets of immunotherapy in some solid cancers (23). So correlation among differential expression genes, PD-L1 and CTLA4 were also evaluated.

### Statistical Analysis

All the statistical analysis were conducted using R (version 3.6.3, www.r-project.org). The log-rank test from survival package (version: 3.1-11) was adopted to analysis the survival differences in the TCGA and CGGA datasets. We perform univariate and multivariate analyses to construct the Cox proportional hazards regression model. We compared the differences of immune infiltration between groups using the Mann-Whitney test. Spearman method was adopted to assess correlations.

## Results

### Establishment of Prognostic Immune-Related Gene Pairs Signature

**Figure 1:**
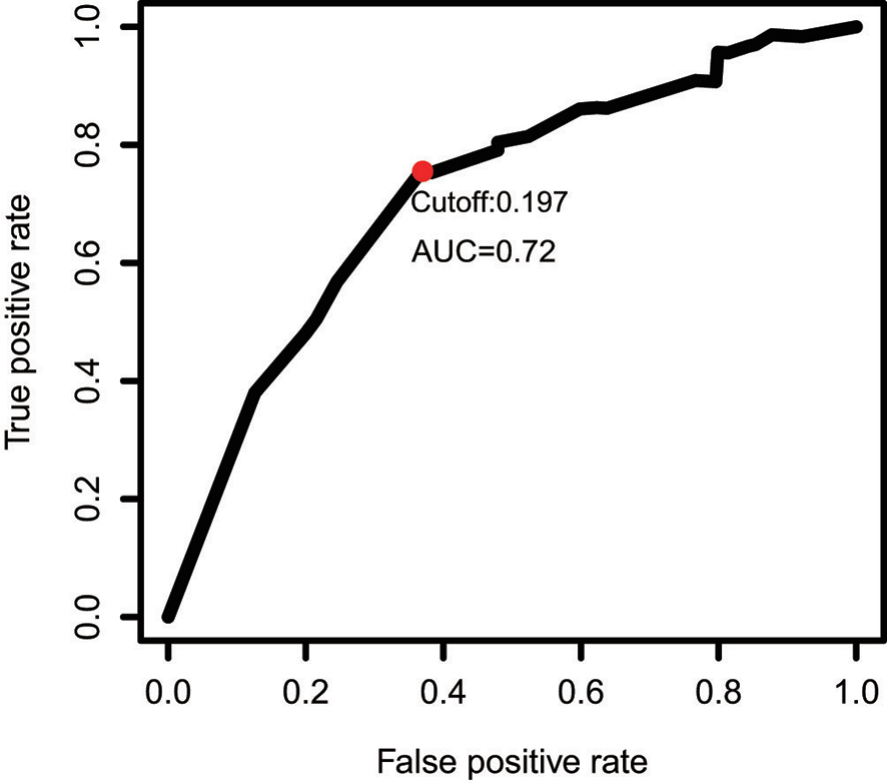
One year time-dependent ROC curve for IRGPI in the TCGA dataset. The optimal cutoff value to differentiate high and low risk groups is 0.197.

**Figure 2:**
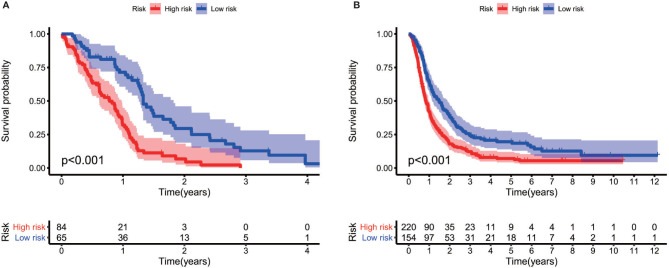
Log-rank test identifies different prognosis between different risk groups both in the TCGA cohort **(A)** and the CGGA cohort **(B)**.

The gene expression profiles of GBM patients in the TCGA dataset (n= 149) were used as the discovery cohort. According to the evaluation criterion that median absolute deviation (MAD)>0.5, the high variation genes were retained as candidate genes. The filtered data used immune-related genes (IRGs) downloaded from the ImmPort database to establish immune-related gene pairs (IRGPs). 56554 IRGPs were conducted from 724 immune-related genes. Screening IRGPs by means of the log-rank test, cox regression and multiple lasso regression, we finally selected 5 IRGPs and calculated immune-related gene pairs index (IRGPI). These 5 IRGPs are composed of 9 unique IRGs, most of which relate to antigen processing and presentation, antimicrobials and cytokines (**Table 1**). Then each GBM patient’s risk score in the training group was operated. Through one year time-dependent ROC curve analysis, the best cut-off value of the IRGPI was 0.197 for criterion to distinguish different risk groups (**Figure 1**). High risk group have a worse prognosis than low risk groups (**Figure 2A**), indicating IRGPI dividing patients significantly. Risk and other clinical factors including age, gender, radiotherapy and chemotherapy were analyzed in the univariate and multivariate Cox analysis (**Table 2**). Risk based on IRGPI signature showed statistical significant in both Cox analysis. **Supplementary Table 1** showed detailed information of each patient including survival time, status, riskscore and risk group in the TCGA dataset.

**Table 1:** Immune-related gene pairs information and coefficients.

IRG1	Category	IRG2	Category	Coef
HSPA6	Antigen Processing and Presentation	BMP2	TGFb Family Member	0.464
PSMC3	Antigen Processing and Presentation	MDK	Cytokines	-0.358
FGF2	Antimicrobials	OSMR	Cytokine Receptors	-0.460
PPP4C	Antimicrobials	MDK	Cytokines	-0.293
LEFTY2	Cytokines	MSTN	Cytokines	0.555

**Table 2:** Univariate and multivariate analysis of prognostic factors in the TCGA and CGGA dataset.

Datasets	Factor	Univariate cox analysis		Multivariate cox analysis	
		HR(95%CI)	P-value	HR(95%CI)	P-value
TCGA	Age	1.02(1.00-1.04)	0.01	1.01(0.99-1.02)	0.44
	Gender	0.92(0.62-1.35)	0.65	0.77(0.49-1.21)	0.25
	Radiotherapy	0.16(0.09-0.27)	3.22E-12	0.14(0.07-0.29)	1.45E-07
	Chemotherapy	0.45(0.30-0.67)	8.72E-05	0.71(0.42-1.22)	0.22
	Risk	2.79(1.87-4.16)	5.27E-07	3.09(2.01-4.74)	2.40E-07
CGGA	Age	1.01(1.00-1.02)	0.03	1.01(1.00-1.02)	0.04
	Gender	0.92(0.74-1.16)	0.49	0.91(0.71-1.15)	0.42
	Radiotherapy	0.69(0.52-0.92)	0.01	0.66(0.49-0.90)	0.01
	Chemotherapy	0.46(0.34-0.61)	9.04E-08	0.49(0.37-0.66)	3.01E-06
	Risk	1.72(1.37-2.16)	2.84E-06	1.70(1.33-2.16)	1.66E-05

### Validation of Prognostic Prognostic Gene Pairs Signature

The gene expression data of GBM patients in the CGGA dataset (n= 374) were used to verify whether IRGPI signature has the same role as that in the TCGA dataset. Different risk groups according to the IRGP signature have distinct prognosis, as similar with the result of training group (**Figure 2B**). Through the univariate and multivariate Cox analysis, risk groups also show significantly independent factor for survival (**Table 2**). Similar items of each patient in the CGGA database were displayed (**Supplementary Table 2**).

### Immune Infiltration Pattern Between Different Risk Groups

**Figure 3:**
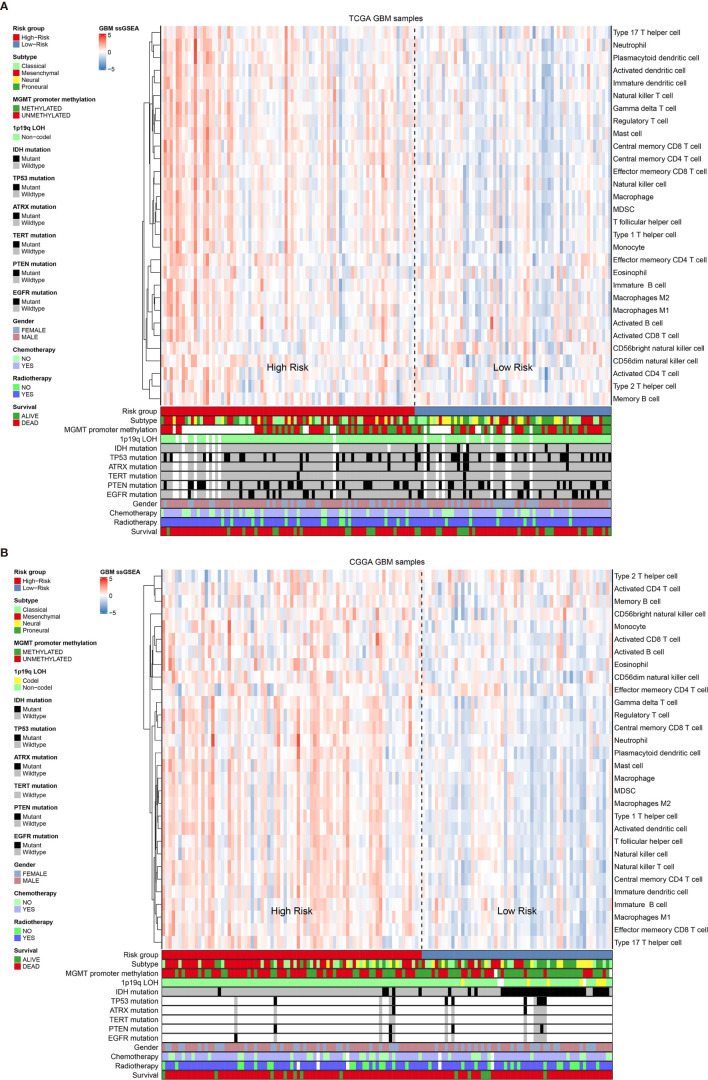
The overall immune cells infiltration associated with Verhaak subtype, some mutations and clinical information in the TCGA dataset **(A)** and the CGGA dataset **(B)**.

**Figure 4:**
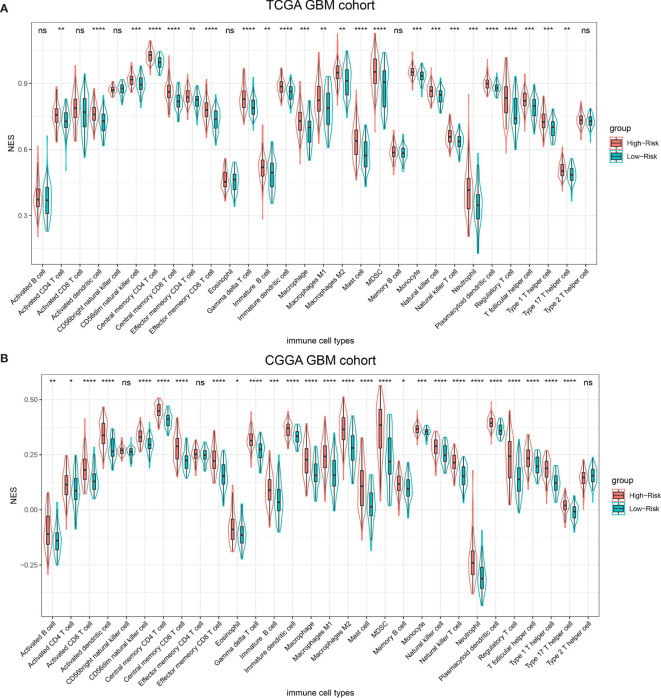
Thirty types of specific immune cells infiltration in the TCGA dataset **(A)** and the CGGA dataset **(B)**. NS, p > 0.05, *p <= 0.05, **p <= 0.01, ***p <= 0.001, ****p <= 0.0001.

Previous publication has elaborated immune infiltration is related to cancer prognosis (24). And single sample gene set enrichment analysis (ssGSEA) has been adopted to assess immune cell infiltration (25). For each patients from different risk groups in the TCGA dataset and a subset from the same platform in the CGGA database, we made use of ssGSEA to calculate the relative abundance of 30 immune cells from 16 immune populations. Based on overall immune infiltration (**Figure 3**), we found that majority of patients in high risk group had higher immune infiltration and belonged to mesenchymal subtype based on Verhaak subtype (26). While in the low risk group, more patients harbored MGMT promoter methylation and IDH mutation and proneural subtype increased, which indicated better prognosis (26–29). Comparison and significant differences of specific immune cells between high and low risk groups have been exhibited (**Figure 4**). Although some immune positive cells including activated CD4 T cell (30), activated dendritic cell, effector memory CD8 T cell, natural killer cell and type 1 T helper cell are higher in the high risk group, more immune repressive cells involving gamma delta T cell (31), macrophage M2 (32), MDSC (myeloid derived suppressor cells) (33), neutrophil (34), regulatory T cell (35) contribute to worse prognosis, similar results from the TCGA and CGGA datasets.

### Functional Pathways Assessment of Different Groups

**Figure 5:**
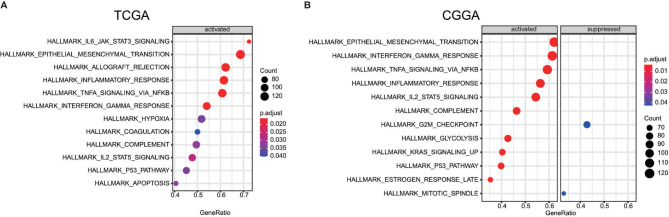
Bubble plot showed some pathway which are more enriched in high risk group than low risk group between the TCGA dataset **(A)** the CGGA dataset **(B)**.

**Figure 6:**
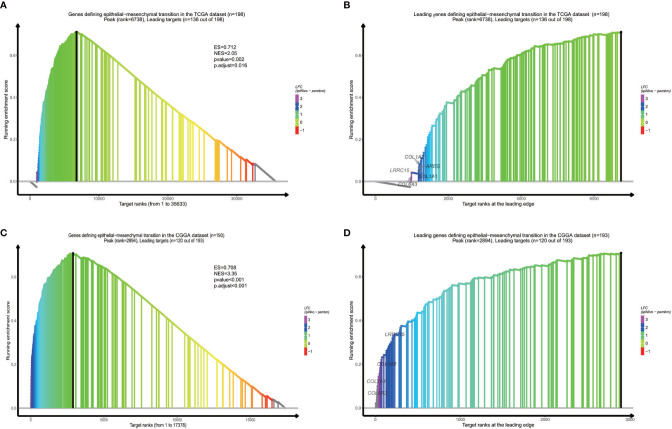
GSEA proves epithelial−mesenchymal transition is activated in high risk group from the dataset **(A)** and the CGGA dataset **(C)**. Some specific genes including COL6A3, COL1A1, COL1A2 and LRRC15 are overexpressed **(B, D)**.

**Figure 7:**
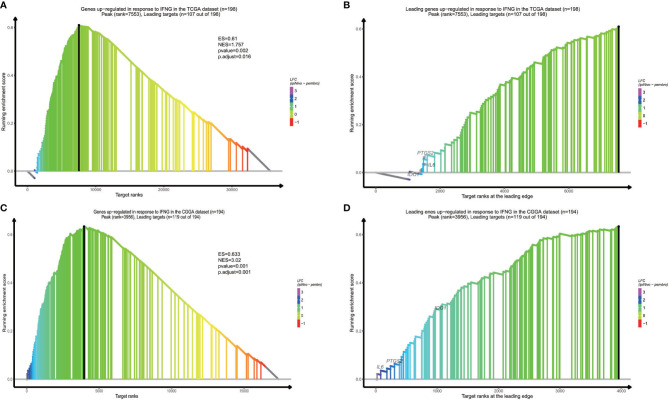
Response to IFNγ pathway result is vibrant in high risk group from the TCGA dataset **(A)**, and the CGGA dataset **(C)**. Overexpressed genes in two datasets contain IDO1, IL6 and PTGS2 **(B, D)**.

In order to find out which pathways change more in high risk group than low risk group, we adopted gene set enrichment analysis (GSEA). The bubble plot showed top twelve pathways enriched in the high risk group from two cohorts (**Figure 5**). Statistic value of these pathways were presented (**Supplementary Tables 3** and **4**). Epithelial-Mesenchymal Transition (EMT), interferon gamma response, IL2/STAT5 signaling and some other pathways are activated in the high risk group, which manifest immune suppression (36), drug resistance (37), tumor evasion (38). Particularly in the epithelial-mesenchymal transition pathway (**Figures 6A, C**), we can see some genes including COL6A3, COL1A1, COL1A2 and LRRC15 express higher in the high risk group (**Figure 6B**), which also can be seen in high risk group from the CGGA dataset (**Figure 6D**). From the response to IFNγ pathway (**Figures 7A, C**), IDO1, IL6 and PTGS2 expressed higher in the high risk group between two cohorts (**Figures 7B, D**).

### Relationship Between Selected Genes and Immune Reaction

**Figure 8:**
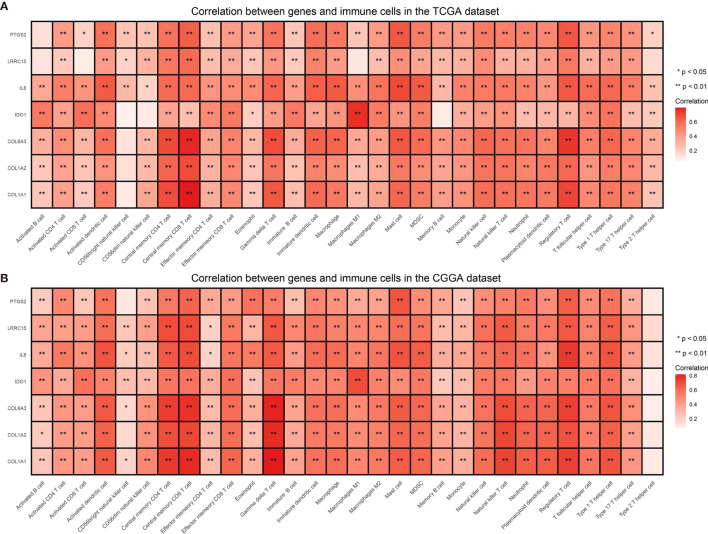
Correlation between selected genes and immune cells from the TCGA **(A)** and the CGGA dataset **(B)**. *P value < 0.05, **P value < 0.01.

**Figure 9:**
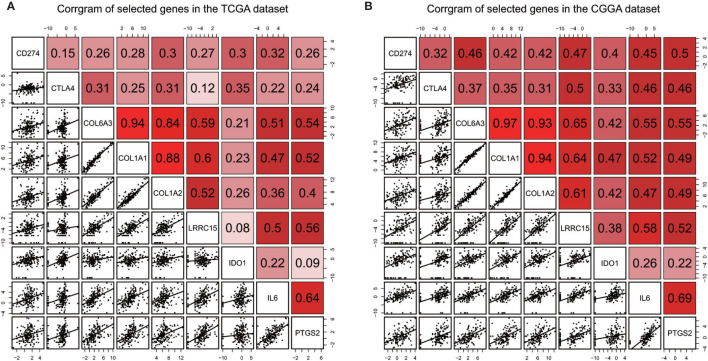
Correlation between selected genes and immune checkpoints CD274/PD-L1, CTLA4 in the TCGA dataset **(A)** and the CGGA dataset **(B)**.

Immune checkpoints such as PD-L1/CD274 and CTLA4 are often overexpressed in tumor cells to devitalize effector T cells and suppress immune responses, which could be targets for immunotherapy (39–41). To determine whether these selected genes have an impact on immune inhibition, they were estimated through their correlation with immune cells and two immune checkpoints. The selected genes were mostly correlated to gamma delta T cell, regulatory T cell, central memory T cell (42), which are immune negative or noneffective cells (**Figure 8** and **Supplementary Figures 1** and **2**). Moreover, all seven genes had positive correlation with PD-L1/CD274 and CTLA4 (**Figure 9** and **Supplementary Tables 5** and **6**).

## Discussion

Glioblastoma multiforme (GBM) is one of the lethal tumor in the central nervous system. The standard treatment includes surgery, radiotherapy, chemotherapy. However, no novel regimen has been introduced into clinical practice to obviously improve survival of GBM patients in recent years. Immune system is one of defensive lines against tumor and immunotherapy has become a research focus among different types of cancer to prolong patients’ survival. The current immunotherapy comprises cell therapy, peptide vaccine, and immune checkpoint inhibitors (33). As for GBM, only less than 10% patients respond to checkpoint inhibition (43). This phenomenon implies advanced malignancies have complex interactions with the immune system. It is urgent to find out new immune related biomarkers to predict prognosis and give each patient personalized treatment.

As for exploring prognostic signatures, it’s difficult to analyze the gene expression profiles of every tumor specimen. Data need to be standardized properly due to different sequencing platforms and tumor samples. In order to avoid technical bias from different platforms, we adopted an new method not require scaling and normalization (44). In this study, we found out an immune-related gene pairs (IRGPs) signature independently predict prognosis. Proneual subtype, IDH mutant and MGMT promoter methylation are well known prognostic factors for longer survival of GBM patients (27, 28, 45, 46). A substantial proportion of the high risk group patients belong to mesenchymal subtype, while proneural subtype increases in the low risk group. Compare with high risk group, more patients in the low risk group have IDH mutation and MGMT promoter methylation. Other factors including EGFR amplification, TERT promoter mutation, TP53 mutation don’t show difference between two groups because limited mutation information of GBM patients in CGGA dataset. Meanwhile, different risk group based on this signature has distinct immune cells infiltration. High risk group has some immune positive cells and more immune suppressive cells, indicating battle between immune cells and tumor cells is a continuous process of elimination, equilibrium, and escape (47). Previous study has shown that macrophage dominated and high M2 macrophage polarization consistent with an immunosuppressed tumor microenvironment, which foreboded a poor outcome (48). In our study, high risk group patients have more M2 macrophage than low risk group patients. It manifests there is a discrepancy of immunosuppressed microenvironment between different groups. Furthermore, we discover epithelial-mesenchymal transition signaling (49), TNFαsignaling *via* NF-κB (50), IL-2/STAT5 signaling (36), interferonγ response signaling and some other pathways are activated in high risk group, most of which trigger tumor cells malignant progression, immune evasion, metastasis and poor prognosis. In two specific pathways (epithelial-mesenchymal transition and interferonγ response), we obtain some genes express higher in high risk group than that in low risk group. Previous article has verified silence of COL6A3 and COL1A2 can inhibit tumor cell proliferation, migration, and invasion in the gastric cancer (51). COL6A3 also has similar role in the colorectal cancer (52). Another studies have reported that COL1A1 is related to facilitate cell invasion in glioma (53). LRRC15 has been confirmed as a immunotherapy resistant target in the single-cell RNA sequencing experiment (54). Experiments showed the combination of IL-6 and PD-1/PD-L1 inhibitors promotes antitumor immunity (55) and PTGS2 deletion sensitized tumors to immunotherapy (56). One research has demonstrated increased levels of IDO1 in the glioblastoma cell had positive correlation with human-infiltrating T cells leading to poor prognosis (57). In murine GBM model, IDO1 inhibition combine with radiotherapy and PD-1 blockade increased survival (58). Most important of all, IDO1 inhibitor could benefit a subset of patients with recurrent malignant glioma in a phase 1 study (59). In our study, we found that these selected genes were most correlated to immune repressive cells and noneffective memory T cells. They also had a positive relationship with CD274 and CTLA4. All these findings indicated that high risk group might be more aggressive and immunosuppressive than low risk group. Immune heterogeneity existed between different risk groups. The mechanism of tumor invasion and immune resistance involving COL6A3, COL1A1, COL1A2, LRRC15, IDO1, IL-6 and PTGS2 need to be further researched aiming to improve prognosis of aggressive glioblastoma.

Our study also have some limitations. First, this signature is based on gene expression profiles, which are not widespread applied owing to expenditure and high requirement of bioinformatics knowledge. In addition, the mechanism of seven genes in different groups has not been explored, though they were potential targets for immunotherapy.

## Conclusion

In conclusion, we identified an immune related gene pairs signature. Different groups based on this signature have distinct prognosis and immune heterogeneity. Some biological processes and genes have indicated the poor prognosis is related to tumor immune evasion, malignant progression, metastasis. The detailed function of these targets need to be explored to correct immune dysfunction and make all patients benefit from personalized immunotherapy.

## Data Availability Statement

Publicly available datasets were analyzed in this study. The TCGA-GBM dataset can be found from TCGA database (https://cancergenome.nih.gov/). The GBM data from CGGA database can be gained in the website (http://www.cgga.org.cn/).

## Ethics Statement

Written informed consent for participation was not required for this study in accordance with the national legislation and the institutional requirements.

## Author Contributions

NZ and MG contributed to the conception of the study. HS, XQ, ZZ, and DL contributed to manuscript preparation. NZ performed the data analyses and wrote the manuscript. MG, TJ, and XP helped perform the analysis with constructive discussions. All authors contributed to the article and approved the submitted version.

## Conflict of Interest

The authors declare that the research was conducted in the absence of any commercial or financial relationships that could be construed as a potential conflict of interest.
