# Supplementary figures and images for: An Immune-Related Gene Pairs Signature Predicts Prognosis and Immune Heterogeneity in Glioblastoma
Source: Front Oncol. 2021 Apr 13;11:592211. doi: 10.3389/fonc.2021.592211 (PMC8076680; doi:10.3389/fonc.2021.592211)

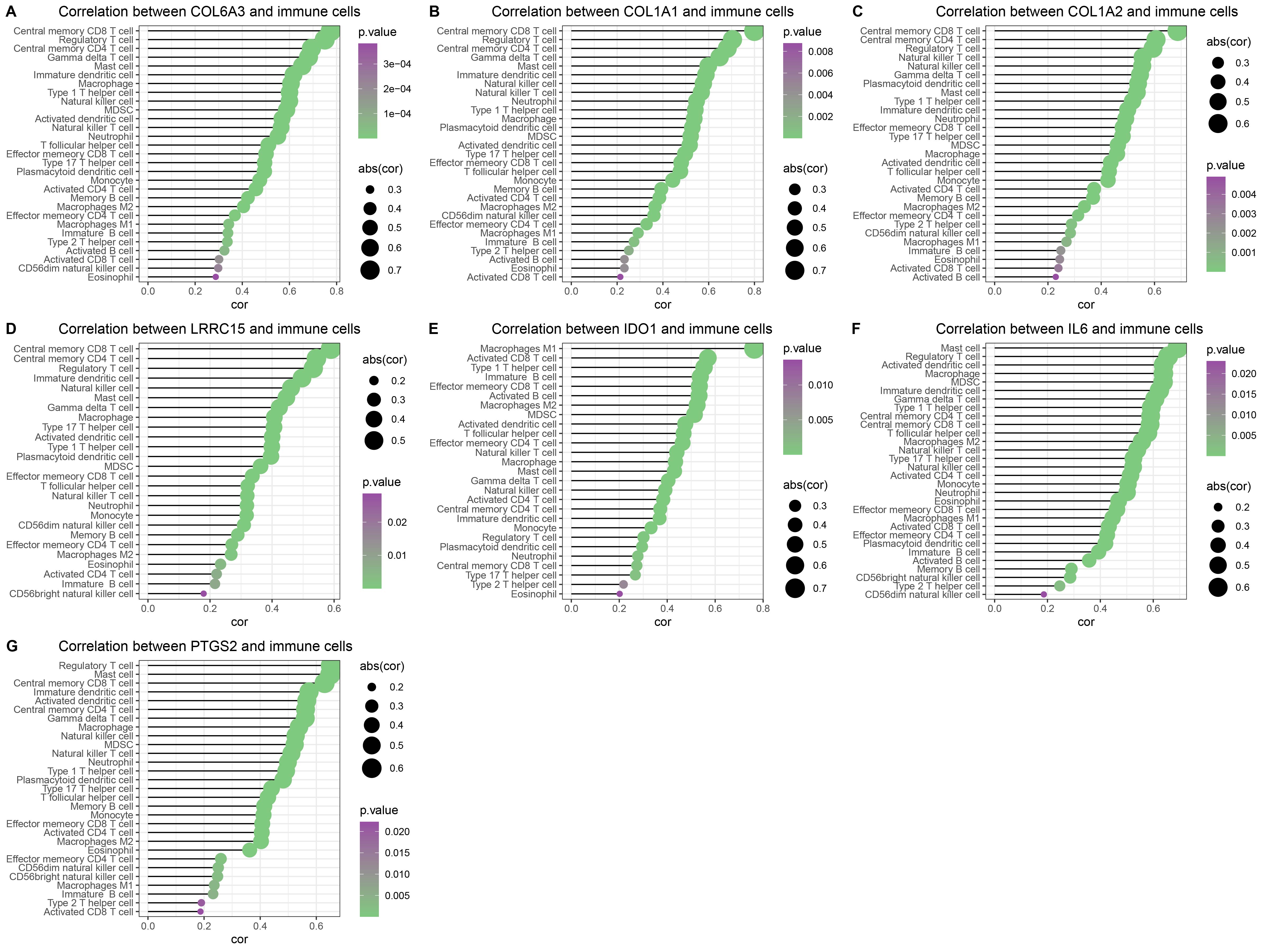

Supplement: Supplementary file 7 [file Image_1.jpeg]

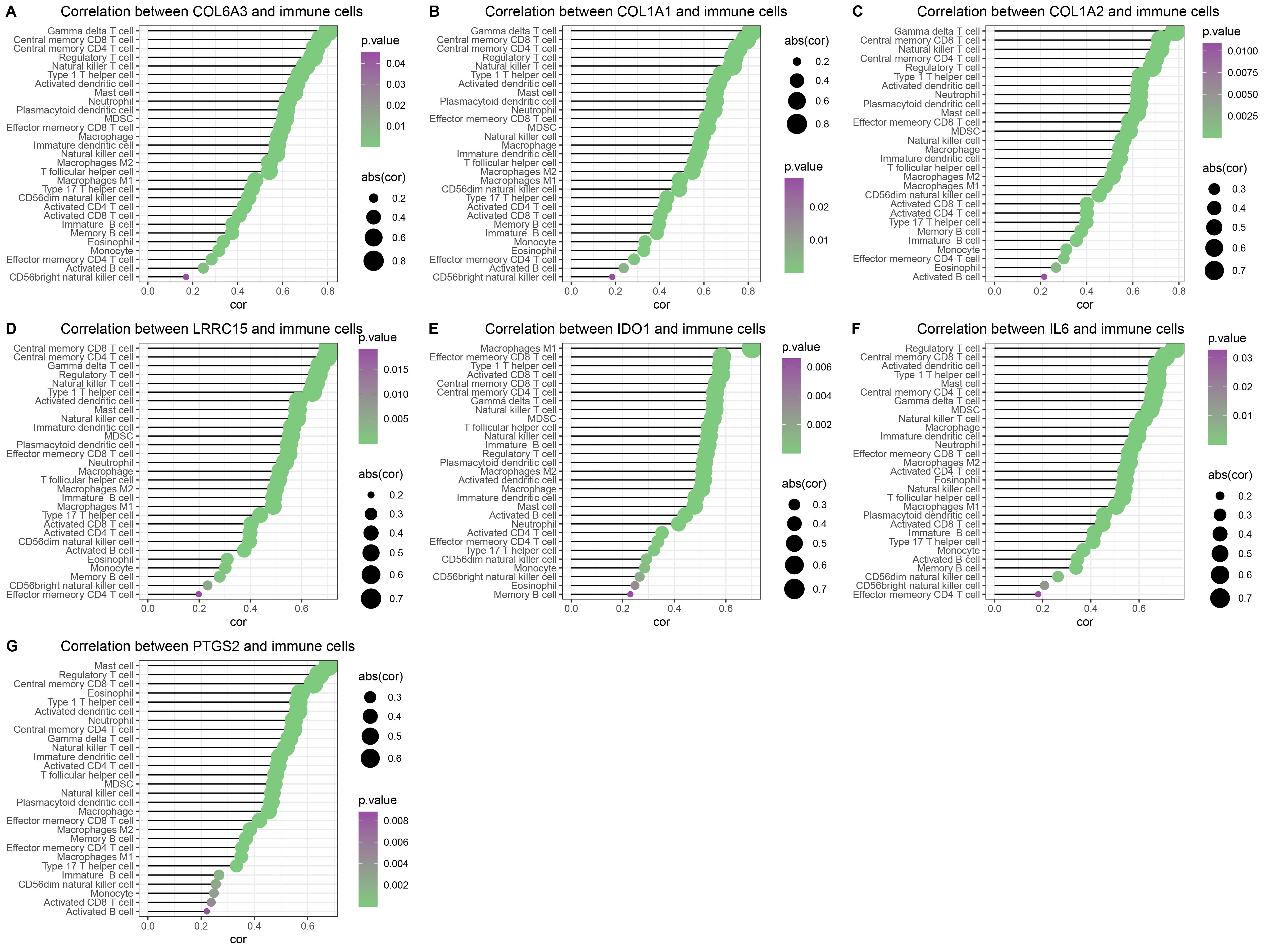

Supplement: Supplementary file 8 [file Image_2.jpeg]
